# Enhancing Range Use in Free-Range Laying Hen Systems: The Impact of Vegetation Cover over Time

**DOI:** 10.3390/ani15091204

**Published:** 2025-04-23

**Authors:** Markus Pacher-Deutsch, Philipp Meyer, Harald Meimberg, Martin Gierus

**Affiliations:** 1 Institute of Animal Nutrition, Livestock Products, and Nutrition Physiology, BOKU University, Muthgasse 11, 1190 Vienna, Austria; markus.deutsch@boku.ac.at; 2 Institute of Integrative Nature Conservation Research, BOKU University, Gregor-Mendel-Strasse 33, 1180 Vienna, Austria; philipp.meyer@boku.ac.at (P.M.); meimberg@boku.ac.at (H.M.)

**Keywords:** free-range, laying hens, outdoor range, vegetation cover, spatial distribution, range use, animal welfare

## Abstract

Outdoor ranges in free-range laying hen systems are often underutilised, likely due to their open, uncovered nature. Given that the domestic chicken’s ancestor inhabits forests with high canopy cover, similar habitat preferences can be expected. To assess the impact of vegetation cover, range use was recorded on two outdoor ranges before and one and two years after planting bushes. A barrier tape grid was placed on each outdoor range, images were taken every 10 min over three days and hens per quadrant were counted. Data were analysed using general additive models. On both outdoor ranges, the number of hens increased in quadrants with newly planted bushes. While hen numbers declined with increasing distance from the shed in all three years, this effect diminished in the two years after planting. Furthermore, hens on both outdoor ranges in all three years showed diurnal patterns in range use, resembling the natural behaviour of their ancestor. The findings support the hypothesis that vegetation cover plays a crucial role in promoting more even range use, including in parts further away from the shed. To encourage natural behaviour and mitigate the negative effects of poor range utilisation, outdoor ranges should incorporate evenly distributed vegetation cover.

## Simple Summary

In free-range laying hen systems, large parts of the outdoor ranges are often used very poorly, raising the question of how much hens actually benefit from them. One reason for this may be found by looking at the ancestor of the domestic chicken—the red junglefowl—which inhabits rather dense forests. Most outdoor ranges, however, are open fields with little or no vegetation. This study examined two outdoor ranges prior to and one and two years after planting bushes by dividing the areas with a barrier tape grid and counting hens in the resulting quadrants. The results showed that after planting bushes, hens increasingly used these areas. Although hens generally preferred staying close to the shed, this effect became weaker in the second and third year, meaning that more hens were willing to explore areas further away from the shed. Also, hens on both outdoor ranges showed diurnal patterns in range use, which matches the natural behaviour of the red junglefowl. The findings suggest that vegetation cover plays a key role in encouraging more even and widespread range use. To support natural behaviour and ensure that hens fully benefit from outdoor access, these areas should include well-distributed vegetation cover.

## 1. Introduction

Compared to cages (which have been banned in EU Member States since 1st January 2012 [1]) or barn systems, free-range systems for laying hens must be provided with an open-air outdoor area (i.e., outdoor range) [2]. However, regulations on the size and design of and access to these outdoor ranges vary around the world. For example, in EU Member States, hens are required to have continuous daytime access to an outdoor range of at least 4 m^2^ per hen.

Most modern, free-range systems have only open fields as outdoor ranges [3]. These systems lack vegetation cover, such as trees or bushes, which are potentially important in meeting the environmental needs of laying hens. To understand what an ideal outdoor range for hens might look like and what environmental features should be present, the habitat of the red junglefowl (*Gallus gallus*) should be considered. As the ancestor of the domestic chicken [4,5], it is found throughout most of Southeast Asia and inhabits lush jungle forests with high canopy cover and dense ground vegetation [6]. This rich environment allows the red junglefowl to seek shelter when resting or preening [7] or in the event of an attacking predator [8].

By providing an environment that more closely resembles their natural habitat, hens are encouraged to follow their natural instincts [9] and can better engage in important natural behaviours like foraging, dustbathing or sunbathing [10,11]. The ability to forage and scratch on an outdoor range can prevent hens from redirecting pecking behaviour towards conspecifics [12], resulting in less frequent injurious feather pecking [13,14]. The ability to dustbathe in an appropriate outdoor environment can improve plumage conditions and, therefore, overall welfare [15] by reducing feather oiliness [16]. Furthermore, hens in free-range systems also tend to have stronger and healthier bones, probably due to the increased activity and bone loading during outdoor exercise, compared to hens in cages [17]. By allowing such natural behaviours and high levels of activity in free-range systems, animal welfare is promoted. As defined by Fraser et al. (1997), animals with good welfare should be able to perform natural behaviour in a species-appropriate environment and should be free from pain, disease and stress [18]. In addition to welfare problems, poor range use can also lead to environmental problems like clustering around the shed [19,20], with potential damage to the pasture [21], and overfertilisation [22,23] and/or higher rates of parasites [24] and pathogens [25].

Many studies in recent decades have documented poor range use associated with a lack of vegetation cover [13,14,26,27,28,29,30,31]. The number of hens outside the shed as well as their distribution on the outdoor range are indicators of good range use [20]. Studies often have a common approach: comparing outdoor ranges with high levels of cover with others with low levels or dividing outdoor ranges into different sections to assess the impact of cover on range use [13,19,26,27,29,30,32]. However, this approach potentially makes it difficult to compare data because conditions on each farm are unique and each flock will interact with the outdoor range provided differently. As a result, other factors that vary between farms, like flock size [33], housing size [19] or pophole size [20], may outweigh the true effect of cover on range use.

The present study therefore used a novel approach to investigate differences in range use by comparing the same outdoor ranges before (Year 0) and one (Year 1) and two (Year 2) years after planting vegetation cover (e.g., bushes). In this way, other factors remained largely unchanged during data collection and the specific influence of cover could be investigated in more detail. It was expected that the presence of the newly planted bushes would have a positive impact on the range use and spatial distribution of the hens.

## 2. Materials and Methods

### 2.1. Data Collection: Study Site and Animals

This study took place on two different outdoor ranges of a commercial free-range laying hen farm in Styria, Austria. Data collection occurred over three consecutive years with varying flocks: Year 0 (2022), Year 1 (2023) and Year 2 (2024). All flocks consisted of the Lohmann Brown Classic breed and were about the same size (i.e., number of hens per flock) and age at the time of data collection.

#### 2.1.1. Outdoor Range 1

**Figure 1:**
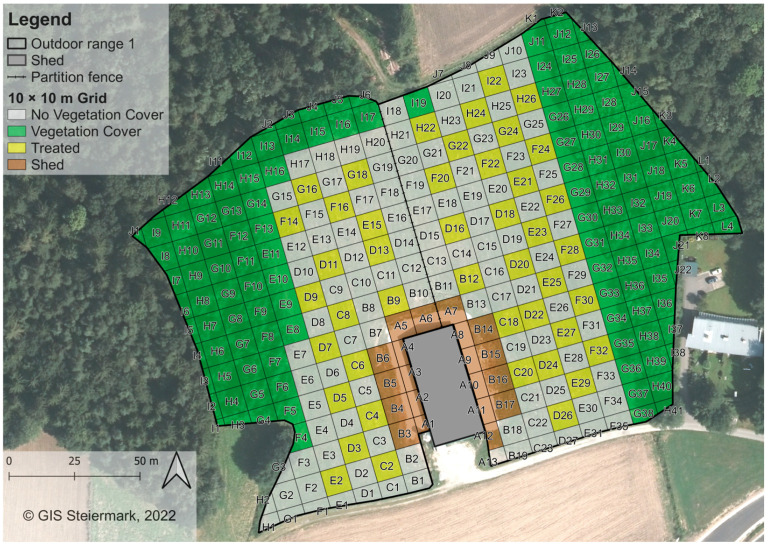
Outdoor range 1 with 10 × 10 m grid. Resulting quadrants were numbered alphabetically and ascended clockwise beginning from the shed. Green quadrants represent quadrants with already existing vegetation cover. Yellow quadrants represent quadrants in which bushes were planted after Year 0. Where possible, bushes were planted every second quadrant to create evenly distributed “islands” with vegetation cover. Brown “shed” quadrants were excluded from statistical analysis. The compass in the lower left corner of the figure points north.

The flocks housed on outdoor range 1 at the beginning of each egg-laying cycle comprised 3000 hens. At the time of data collection in Year 0 (2022), Year 1 (2023) and Year 2 (2024), the flocks comprised approximately 2800, 2800 and 2600 hens due to losses from predators and diseases that were 67, 37 and 69 weeks old, respectively. The total area of outdoor range 1 was approximately 3.0 ha. The shed was located in the centre of the outdoor range (Figure 1). Hens were granted access to the outdoor range through the wintergarden by four popholes on the eastern and four popholes on the western side of the building (eight in total, each 2.9 m in width). The distance between popholes located on the same side of the building was also 2.9 m. During data collection, the popholes opened automatically at 7:50 a.m. each day and were closed after 8:00 p.m. after the farmers had ensured that all hens had returned to the shed.

Most parts of the outdoor range were lacking in vegetation cover in Year 0, except for the eastern and western outer borders of the outdoor range. These parts were characterised by naturally grown pine wood (western side) and an artificially planted mixed forest mainly consisting of poplar and lime trees (eastern side, Figure 1).

#### 2.1.2. Outdoor Range 2

**Figure 2:**
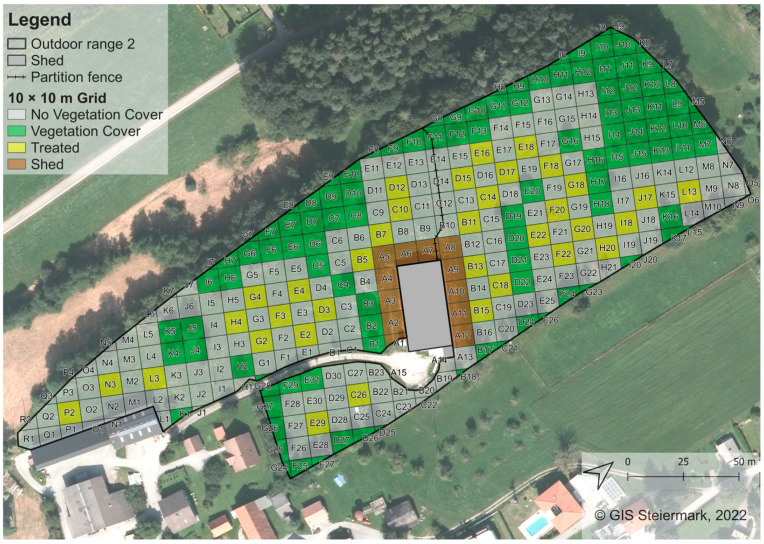
Outdoor range 2 with 10 × 10 m grid. Resulting quadrants were numbered alphabetically and ascended clockwise beginning from the shed. Green quadrants represent quadrants with already existing vegetation cover. Yellow quadrants represent quadrants in which bushes were planted after Year 0. Bushes were planted every second quadrant where possible to create evenly distributed “islands” with vegetation cover. Brown “shed” quadrants were excluded from statistical analysis. The compass in the lower right corner points north.

The flocks housed on outdoor range 2 at the beginning of each egg-laying cycle comprised 2560 hens. At the time of data collection in Year 0 (2022), Year 1 (2023) and Year 2 (2024), the flocks comprised approximately 2500, 2400 and 2500 hens due to losses from predators and diseases that were 51, 54 and 52 weeks old, respectively. The total area of outdoor range 2 was approximately 2.6 ha. The shed was also located in the centre of the outdoor range (Figure 2). Hens had access to the outdoor range through the wintergarden by two popholes on the northern and two popholes on the southern side of the building (four in total, each 4.44 m in width). The distance between popholes located on the same side of the building was 4.5 m. As for outdoor range 1, during data collection, popholes were opened automatically at 7:50 a.m. each day and closed after 8:00 p.m. after the farmers had ensured that all hens had returned to the shed.

Most parts of the northern and western borders of the outdoor range were already covered with a mixed forest, mainly consisting of pine and beech trees. Additionally, scattered fruit trees and bushes (e.g., apple, walnut, lilac, hazel) were already located on small parts of the outdoor range (Figure 2).

#### 2.1.3. Study Design

In this study, the same study design as described in [26] was used and is described in the following. Digital maps of the outdoor ranges were created using the open-access software QGIS version 3.12.1-București [34] with the required geodata provided by the Provincial Government of Styria (Austria), Department 17, State and Regional Development, Statistics and Geo-Information. Based on the shape of the outdoor ranges and the desire to show the space use in greater detail compared to the previous study, grids of 10 × 10 m were laid out on the ground of the outdoor ranges, using barrier tape (Kayser Systems, Rollingen, Luxemburg). This resulted in 299 digital quadrants for outdoor range 1 (Figure 1) and 310 digital quadrants for outdoor range 2 (Figure 2). Each quadrant was assigned a unique ID and characterised by certain attributes such as “cover” (yes = vegetation cover already existed, no = no vegetation cover, treated = planted with bushes after Year 0), “distance” (to the shed in m) or “size” (of the quadrant in m^2^).

Data for outdoor range 1 were collected in April 2022, September 2023 and May 2024, while data for outdoor range 2 were collected in May 2022, June 2023 and May 2024. Data collection dates varied slightly each year due to avian influenza (bird flu) regulations in Austria. Data collection only took place during sunny or cloudy weather; during rainy days, data collection was paused.

The red and white striped barrier tape used for the grid was 8 cm wide, made of highly resistant polyethylene and groundwater neutral. It was brought out on the outdoor ranges one day prior to the beginning of data collection to allow the hens to adapt to it. However, it cannot be ruled out entirely that some hens may have been affected in their ranging behaviour by the tape. For example, despite the fact that it was placed as close as possible on the ground, there was still the possibility of the tape flapping in the wind, potentially discouraging some hens from crossing it. The barrier tape grid quadrants resulted in 197 quadrants for outdoor range 1 (Figure S1 in the Supplementary Materials) and 249 quadrants for outdoor range 2. The different numbers of digital and actual quadrants occurred because the grids were not laid out on the forest parts of the outdoor ranges. Therefore, these digital quadrants did not physically exist on the actual sites and were excluded from data collection.

To cover the relevant parts of the outdoor ranges and every quadrant resulting from the grids, 22 action cameras of the model YI 4K (YI Technology Inc., Shanghai, China) were used for outdoor range 1 and 27 were used for outdoor range 2 (Figure S4). Every camera took a digital image every 10 min from 8:00 a.m. to 8:00 p.m. for three days, resulting in 4818 (73 × 22 × 3) digital images for outdoor range 1 and 5913 (73 × 27 × 3) digital images for outdoor range 2 for each year.

On both outdoor ranges, bushes were planted at the end of August in 2022, after the data collection of the first observation year was finished. On outdoor range 1, bushes were planted in 44 quadrants in every second quadrant in groups of 4 (Figure 1). On outdoor range 2, bushes were planted in 35 quadrants, also in groups of 4 (Figure 2). Regional species of bushes such as field maple (*A. campestre*), dog rose (*R. canina*), hazel (*C. avellana*), privet (*L. vulgare*), red dogwood (*C. sanguinea*), wayfaring tree (*V. lantana*) and black elder (*S. nigra*) were chosen not only due to their resilience and growth rate but also to support local biodiversity. Bushes were between 1 m and 1.50 m high at the time of planting.

### 2.2. Data Evaluation

To count hens per quadrant, an automated system using deep learning and image processing was developed. A fine-tuned YOLO (You Only Look Once, Version 8.0.0) model [35] analysed the digital images taken by the cameras by identifying hens and assigning them to predefined grid areas. Grid coordinates were extracted and converted, while timestamps from the metadata of the images enabled temporal tracking. To improve detection accuracy, images were divided into smaller sections and data augmentation techniques were applied. The results were transferred into a CSV file and visualised with annotated images showing detected hens and grid assignments. A detailed explanation of the method is provided in Appendix A.

Vegetation growth was not quantitatively assessed. The various planted bush species exhibited different growth rates depending on factors like sun and wind exposure and water availability. While most bushes grew minimally in the first year, their sheer presence appeared to enhance spatial distribution and range use, a trend confirmed by the farmers. By the second year, most bushes had nearly doubled in size, with only a few replacements needed due to plant mortality.

### 2.3. Heatmaps

To visualise the spatial distribution of hens for each of the three observation years on both outdoor ranges, heatmaps showing the mean number of hens per quadrant were created using R packages “dplyr” (Version 1.1.4) [36], “ggplot2” (Version 3.5.1) [37] and “sf” (Version 1.0-18) [38,39]. To show potential diurnal changes in ranging behaviour, heatmaps were divided into four times of day (i.e., morning (8.00 a.m. to 11:00 a.m.), midday (11:00 a.m. to 2:00 p.m.), afternoon (2:00 p.m. to 5 p.m.), evening (5:00 p.m. to 8:00 p.m.)). A square root transformation was applied to the colour scales to enhance the differentiation of smaller values, making low-value variations more discernible. The analysis of the heatmaps was descriptive in nature, based on the varied colouring of the quadrants across observation years and times of day.

### 2.4. Statistical Data Analysis

Quadrant-specific data were added to the CSV files generated during the digital data evaluation process. These files were then used for statistical analysis and graphical visualisation in R (R 2024 version 4.4.1, Vienna, Austria) [40]. To examine the effects of cover, observation year and distance to the shed on the number of hens per quadrant, generalised additive models (GAMs) with a negative binomial distribution were applied separately for each farm using the “mgcv” package (Version 1.9-1) [41]. Negative binomial distribution was chosen due to the presence of overdispersion in the data, where the variance exceeded the mean. As this study did not refer to the total number of hens per flock but only counted the varying number of hens in the corresponding quadrants throughout observation years, the number of hens per flock was not included in the models.

The models included cover (yes, no, treated), distance to the shed (in meters) and observation year (Year 0 = 2022, Year 1 = 2023, Year 2 = 2024) as parametric terms, along with their pairwise interactions. Time and temperature (in °C) were included as smoothing terms. To account for spatial dependence among quadrants, a Markov random field smoother [41] was applied, with the discrete spatial information being provided by the neighbourhood structure of the quadrants. Since some quadrants along the fence were smaller than the maximum possible size of 100 m^2^, the logarithmic quadrant size was included as an offset term. Model selection for each farm was performed using AIC backwards selection, where potentially non-essential parametric terms and interactions were removed in order to improve model quality. To visualise the marginal effects of significant explanatory variables and interactions, the package “marginaleffects” [42] (Version 0.25.0) was used.

## 3. Results

### 3.1. Outdoor Range 1

On outdoor range 1, the total number of 197 quadrants comprised 105 quadrants with no cover during the whole data collection period (cover = no), 28 quadrants with already existing vegetation cover (e.g., trees or bushes grown prior to data collection; cover = yes), 44 quadrants in which bushes were planted after Year 0 (2022, cover = treated) and 20 quadrants close to the shed (cover = shed). “Shed” quadrants were excluded from statistical analysis as they were defined as uncovered quadrants within 10 m distance from the shed (quadrants A1–A12, B3–B6 and B14–B17), an area inherently characterised by high hen densities. Including these quadrants probably would have introduced a structural bias by distorting the effect of “cover = no”. The estimated effect of this category would likely have been overestimated as it would have falsely appeared that uncovered quadrants generally lead to a higher number of hens, when in fact the shed’s proximity was the primary factor responsible for the higher number of hens in these quadrants. These considerations applied to both outdoor range 1 and outdoor range 2. The distance to the shed of the quadrants included ranged from 14 m to 117 m (measured from the centre point of each quadrant).

The estimated model coefficients and their *p* values for the final model of outdoor range 1 as well as the approximate significance level of the smooth terms are presented in Table 1. AIC backwards selection showed that the best model included the parametric effects of cover, distance to the shed and observation year, as well as the interaction of cover and year, cover and distance, and year and distance (for the full model formular of outdoor range 1, see Appendix B).

**Figure 3:**
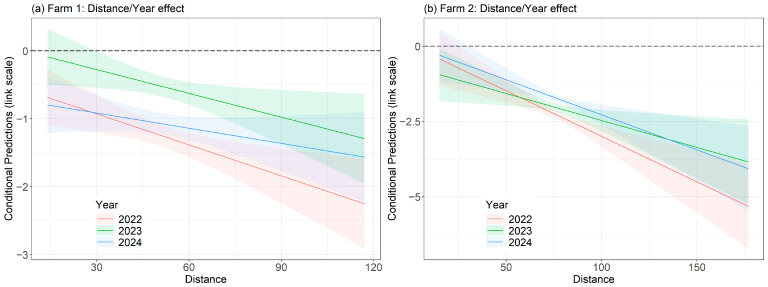
(**a**) Effect plot of the interaction of the distance to the shed and year for outdoor range 1. (**b**) Effect plot of the interaction of the distance to the shed and year for outdoor range 2. The values/factors of Q.Nr (Quadrant ID), time and temperature were excluded from predictions; other parameters were set to their mean/mode values. Different x-axes resulted from different maximum distances to the shed for outdoor range 1 and outdoor range 2. The y-axes differ because the default settings of the R package for the maximum visualisation of the results were retained. Conditional predictions (link scale) refer to model-based predictions on the level of the link function used. The dashed line marks the baseline.

To interpret the results, the significance level was set to *p* < 0.05. The output of the final model revealed that there were no significant differences in the number of hens in covered (cover = yes; *p* = 0.995) or treated quadrants (*p* = 0.099) compared to uncovered ones (cover = no) in 2022 (Table 1). In 2023 (Year 1) and 2024 (Year 2), the number of hens was significantly higher in treated quadrants (*p* < 0.05) and significantly lower in covered quadrants (cover = yes; *p* < 0.05) compared to such quadrants in 2022 (Table 1). In 2023 (Year 1), there were significantly more hens in quadrants without cover (*p* < 0.05), whereas the number of hens in such quadrants decreased again in 2024 (Year 2; *p* < 0.05). The number of hens decreased with increasing distance to the shed (*p* < 0.05) in all three categories of quadrants in 2022 (Year 0), while this negative association weakened significantly in 2023 (Year 1; *p* < 0.05) and 2024 (Year 2; *p* < 0.05, Table 1, Figure 3a). Smooth terms for Temperature and Time by Year are shown in the Supplementary Materials (Figure S2).

### 3.2. Outdoor Range 2

On outdoor range 2, the total number of 249 quadrants comprised 153 quadrants with no cover during the whole data collection period (cover = no), 49 quadrants with already existing vegetation cover (e.g., trees or bushes grown prior to data collection; cover = yes), 35 quadrants in which bushes were planted after the first year of data collection in 2022 (cover = treated) and 12 quadrants close to the shed (cover = shed). As for outdoor range 1, “shed” quadrants again were excluded from statistical analysis (quadrants A1–A12). The distance to the shed of the quadrants included ranged from 15 m to 177 m (measured from the centre point of each quadrant).

The estimated model coefficients and their *p* values for the final model of outdoor range 2 as well as the approximate significance level of the smooth terms are presented in Table 2. AIC backwards selection showed that the best model included the parametric effects of cover, distance to the shed and observation year, as well as the interaction of cover and year, cover and distance, and year and distance (for the full model formular of outdoor range 2, see Appendix B).

There were significantly more hens in covered quadrants (cover = yes; *p* < 0.05) compared to uncovered quadrants (cover = no) in 2022 (Year 0; Table 2), while no such significant difference occurred between uncovered and treated quadrants (at this time, treated quadrants were also uncovered, bushes were planted after the data collection in 2022 (Year 0; *p* = 0.068, Table 2). In 2023 (Year 1) and 2024 (Year 2), the number of hens in uncovered quadrants was significantly lower than in 2022 (*p* < 0.05, Table 2). In 2024, there were significantly more hens in covered quadrants (*p* < 0.05) as well as in treated quadrants (*p* < 0.05) compared to such quadrants in 2022 (Table 2). However, for covered (*p* = 0.607) and treated (*p* = 0.645) quadrants, no such effect was recorded in 2023 (Table 2). The number of hens decreased with increasing distance to the shed (*p* < 0.05) in 2022. However, this negative association effect was significantly weakened in 2023 (Year 1; *p* < 0.05) and 2024 (Year 2; *p* < 0.05, Table 2, Figure 3b). Smooth terms for Temperature and Time by Year are shown in the Supplementary Materials (Figure S3).

### 3.3. Heatmaps

#### 3.3.1. Outdoor Range 1

**Figure 4:**
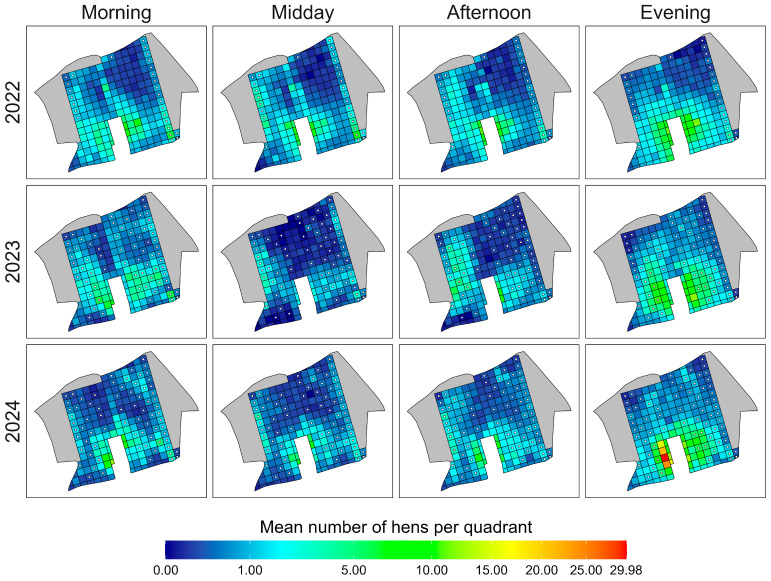
Heatmaps of spatial distribution patterns of hens on outdoor range 1. Shown are separate heatmaps for four different times of day (morning: 8:00 a.m.–11:00 a.m., midday: 11:00 a.m.–2:00 p.m., afternoon: 2:00 p.m.–5:00 p.m., evening: 5:00 p.m.–8:00 p.m.) for each of the three observation years (2022, 2023, 2024). Grey areas were already covered with dense forest and hence were excluded from data collection. Quadrants marked with a black circle with a white filling symbolise quadrants that were already covered (cover = yes) or newly planted with bushes (cover = treated, after 2022).

To visualise the spatial distribution of hens on outdoor range 1 during different times of the day (i.e., morning, midday, afternoon, evening), the mean numbers of hens per quadrant and observation year were used to create heatmaps, as seen in Figure 4.

The distribution patterns of hens varied throughout the day in all three years of data collection. The highest mean number of hens and the most extensive range use occurred during the morning and evening hours each year. In 2023 and 2024, more hens ventured into distant areas of the outdoor range, weakening the negative association between distance from the shed and hen numbers (Table 1). The lowest mean numbers of hens and the most limited distributions were recorded at midday in 2023.

In 2022, before the bushes were planted, few hens used the most distant, vegetation-free northern area (to the right when facing the shed). However, in 2023 and 2024, more hens visited this area, particularly in the morning and evening. The most even and widespread range use was observed in the evenings of these two years (Figure 4).

#### 3.3.2. Outdoor Range 2

**Figure 5:**
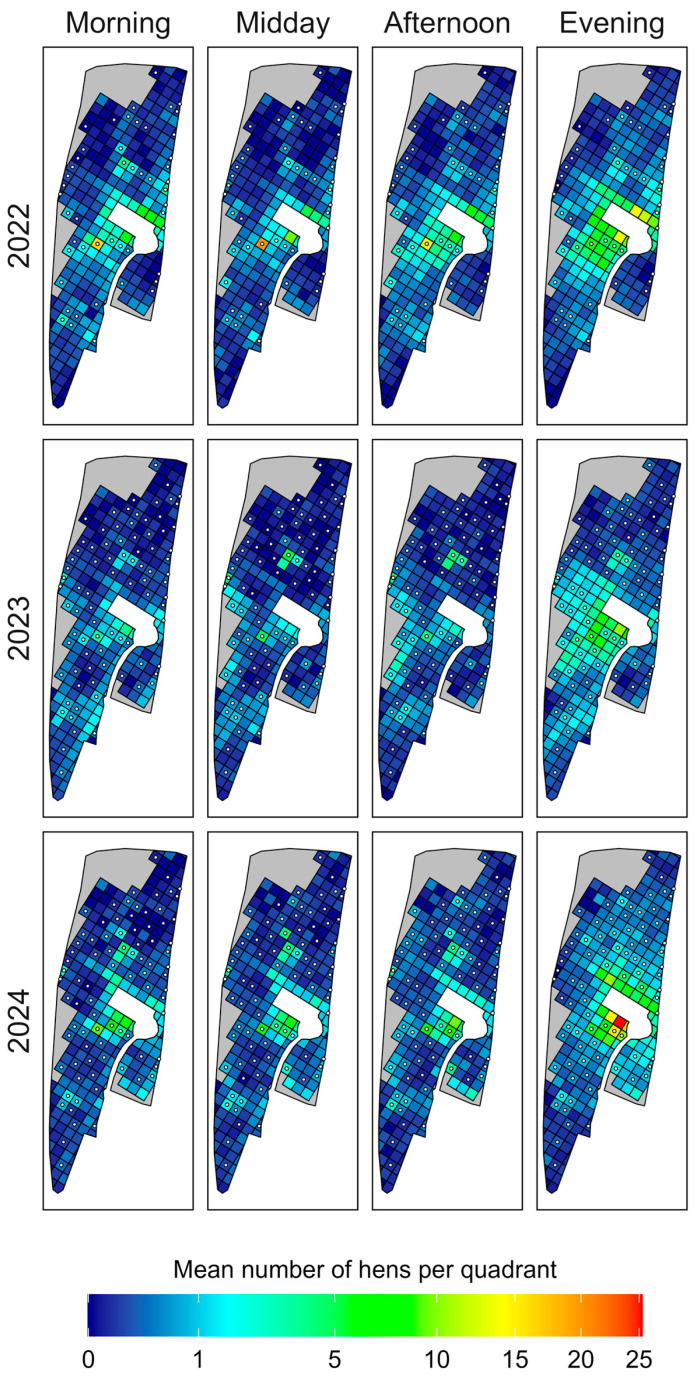
Heatmaps of spatial distribution patterns of hens on outdoor range 2. Shown are separate heatmaps for four different times of day (morning: 8:00 a.m.–11:00 a.m., midday: 11:00 a.m.–2:00 p.m., afternoon: 2:00 p.m.–5:00 p.m., evening: 5:00 p.m.–8:00 p.m.) over the three observation years (2022, 2023, 2024). Grey areas were already covered with dense forest and hence were excluded from data collection. Quadrants marked with a black circle with a white filling symbolise quadrants that were already covered (cover = yes) or newly planted with bushes (cover = treated, after 2022).

To visualise the spatial distribution of hens on outdoor range 2 during different times of the day (i.e., morning, midday, afternoon, evening), the mean numbers of hens per quadrant and observation year were used to create heatmaps, as seen in Figure 5.

Distribution patterns did not show major changes throughout the day, except for the evening hours, where the outdoor range was used most evenly in all three years. In 2023, hens in general preferred the southern part of the outdoor range over the northern part. As on outdoor range 1, most hens were recorded near the shed, with a decreasing number with increasing distance to the shed. However, in 2023 and 2024, more hens also visited former uncovered quadrants, leading to a better overall spatial distribution and a weakening of the distance effect. Notably, during the whole day in all years, hens visited already covered quadrants quite frequently (Table 2), independent of their distance to the shed. During evening hours, hens also made greater use of uncovered quadrants. In 2024, more hens visited the small eastern part of the outdoor range compared to the previous years. Hens were distributed the most evenly in the evening hours in 2024 (Figure 5).

## 4. Discussion

This study investigated the impact of vegetation cover on the range use of free-range laying hens. Therefore, the number of hens before (2022, Year 0) and one (2023, Year 1) and two (2024, Year 2) years after the planting of bushes in previously uncovered areas was compared. In both investigated outdoor ranges, the number of hens increased in Year 2 of data collection in areas that were newly planted with bushes. For outdoor range 1, this effect began from Year 1 onwards.

Uncovered quadrants of outdoor range 1 were visited more frequently in Year 1 but less frequently in Year 2. Some of these quadrants were adjacent to the fence of the outdoor range, which may explain the initial increase in the number of hens in Year 1, followed by a decrease in 2024. Artificial vertical structures, such as fences, can increase space utilisation by allowing hens to move along these structures [26,43]. By Year 2, the newly planted bushes may have reached a certain threshold height, which reduced the attractiveness of the fence and led to a further decline in the number of hens in such quadrants. The number of hens in uncovered quadrants in outdoor range 2 declined in both years after the bushes were planted. This may have been because there were already other well-covered areas at various distances from the shed. The newly planted bushes may have attracted more hens to these areas [8]. Vegetation cover serves as protection from several outdoor environmental factors, such as intense sunshine [3,20], rain or wind [44]. This provides dry, shaded and safe areas where hens can engage in comfort behaviours such as dustbathing or resting [27]. The more such areas are present in outdoor ranges, the more evenly hens can be distributed [31], not only in covered areas, but also in adjacent uncovered areas, as hens would have several refuges throughout the outdoor range where they could seek shelter, including from predators [32,45]. This could encourage hens to move further away from sheds [26,43,46]. Conversely, vegetation cover can also facilitate hunting by providing cover for terrestrial predators or landing sites for avian predators [24]. On outdoor range 1, quadrants already covered by vegetation were visited less frequently after the planting of the bushes. This may be an indication of the importance of the design and type of vegetation cover provided. As for outdoor range 2, the already covered areas consisted of large, scattered fruit trees with large canopies. On the other hand, the type of vegetation offered in outdoor range 1 consisted of very dense poplar and lime trees. This composition may have led to a high number of hens in these areas in the first year when no other vegetation cover was available. However, in Year 1 and Year 2, the treated quadrants were visited more frequently, suggesting that hens may prefer the diverse composition of groups of small bushes combined with fresh grass to the inaccessible areas of poplar and lime trees with less attractive ground vegetation. The effects of different types of vegetation were also documented in several other studies [19,28,29]. Another possible explanation is that, as in outdoor range 1, existing vegetation was only found at distances greater than 50 m from the shed. In contrast, some of the newly planted quadrants were located closer than 50 m. Therefore, these areas may have been more attractive to the hens not only because of their vegetation composition but also because of their closer proximity to the shed.

### Time of Day

The results of this study further suggest that the diurnal pattern of the domestic chicken’s foraging behaviour is similar to that of its ancestor, the red junglefowl. It prefers the proximity of its roost and covered and shaded areas during the day to avoid heat and predators in its natural environment [6]. In commercial systems, the shed is like the roost, so many hens tend to stay close to it [21,22]. This probably explains the fact that, as documented in several other studies [26,28,32,47] and the present study, the number of hens decreases with increasing distance from the shed. However, this pattern appears to be influenced by the availability of vegetation cover and other enrichment structures [3,19,46,48,49]. During the midday and afternoon hours, certain areas of the outdoor ranges were preferred over others. These areas were predominantly closest to the shed, but also included areas with already high levels of vegetation cover. This was particularly pronounced on outdoor range 2, where there were already scattered, well-covered areas at various distances from the shed. These areas were preferred over uncovered areas during these times of the day in all three years of data collection. This correlates with a study by Larsen et al. [29], as they found more hens in distinct outdoor environments with high canopy cover, especially during the midday hours.

In the morning and evening hours, hens on both outdoor ranges moved farther away from the shed and dispersed more, likely reflecting instinctive foraging behaviour like that of the red junglefowl [6]. These findings are consistent with several other studies investigating diurnal patterns of range use [3,26,27,45,46,50]. Savory et al. (1978) found that domestic chickens living in a wild environment foraged most in the evening, possibly to fill their crops with food to survive the night [51]. In addition, the number of hens and their spatial distribution increased during these hours in areas further away from the shed in Year 1 and Year 2. This attenuated the negative association effect between distance to the shed and number of hens in both outdoor ranges. The concept of contrafreeloading, whereby animals prefer to work for food even when it is freely available [52], may explain this behaviour. Although this tendency appears to be reduced in modern breeds of laying hens compared to their wild ancestors [53], it persists to some extent. The increased vegetation cover in the outdoor ranges during these years may have been beneficial in promoting such complex behaviours by providing not only continuous shelter but also additional foraging opportunities, such as young leaves, seeds and insects.

Although the hens used more of the outdoor ranges during the evening hours, the areas closest to the shed were still preferred over others. The shed of outdoor range 1 created a large shadow on the eastern side during sunset, which may have attracted hens through the combination of shade and warm air. In contrast, the low-setting sun on the western side may have deterred some hens by shining directly into the popholes, while others were observed sunbathing in front of them. When the sun disappeared behind the western woods, more hens entered the outdoor range and spread out. In outdoor range 2, such directional light effects were less pronounced near the shed because the popholes faced north and south. However, shadows cast by the setting sun (particularly from the western forest) also appeared to influence hen distribution, as more birds were observed in shaded areas as the sun set. In addition, hens were frequently observed resting or sunbathing on warm gravel near the popholes on both sides of outdoor range 2 during these hours.

Improved outdoor range design in terms of vegetation cover promotes a more even spatial distribution of hens, which can benefit not only animal welfare but also the environment. Hens can engage in natural behaviours [9], which, in turn, can minimise redirected, harmful behaviour towards conspecifics [12], ensuring better health and preventing frustration [54]. Improved spatial distribution leads to more uniform foraging behaviour, resulting in better dispersal of faeces and nutrients. This can reduce the risk of overfertilisation [22,23] and long-term damage to grassland [21], which is desirable for both hens and farmers.

However, in addition to vegetation cover, there are many other farm management-related factors that influence range use, like flock size [13,33,45], housing size [19] or age [3] and breed [55,56] of the flock. In addition, several factors intrinsic to outdoor ranges such as seasons [57,58] or weather conditions [3,20,32] may also influence range use. This study did not investigate differences between sunny and cloudy weather. However, other studies have found more hens outside the shed when the sky was overcast [27,59], probably due to a perceived sense of security or subjective perception of cover [32]. All these factors are likely to influence qualitative and quantitative range use in an overly complex way. The aim of this study was to isolate the factor “vegetation cover” as much as possible and investigate its effect on range use when other factors (e.g., flock size, housing size, shape of the outdoor range, distance to the shed or pophole size) were kept largely constant. Environmental factors like different seasons could be better considered by extending the data collection periods or assessing range use in different weather conditions. This study only investigated range use on a small number of farms. A larger number of outdoor ranges may have been beneficial to support the results. Nevertheless, based on the results of this study, the design of outdoor ranges in free-range poultry systems should focus on the amount and distribution of vegetation cover and the maximum distance from the shed to ensure sufficient range use.

## 5. Conclusions

This study demonstrated the effect of vegetation cover on range use and what impact it may have when newly provided on formerly mostly uncovered outdoor ranges. Two years after the planting of new bushes, hens on both investigated outdoor ranges showed a preference for the areas with newly planted bushes, leading to a more even and widespread spatial distribution. Since the bushes were still rather small and young at the time of the second year, further data collection after a certain time (e.g., in five or ten years) would be of great interest to assess potential long-term changes in range use.

Based on these findings, the optimisation of range use and the provision of necessary resources to ensure better spatial distribution should be a top priority for farmers. Consideration should be given to arrange newly planted vegetation cover in ways that ensure evenly distributed shelter areas for the hens while also allowing farmers to efficiently manage and maintain the quality of their land. This would ensure that hens can follow their natural drives in an outdoor environment that remains intact, promoting comfort behaviour and reducing stress due to predation and extreme weather conditions.

## Figures and Tables

**Table 1:** Estimated model coefficients for the model with number of hens per quadrant as dependent variable for outdoor range 1 (residual df = 115,950) [26].

**Fixed Effects**	**Factor Level**	**Estimate**	**Std. Error**	***p*-Value**
Intercept	-	0.761	0.271	0.779
Cover (Ref. Level: Cover No)	Cover Treated	−0.225	0.136	0.099
Cover (Ref. Level: Cover No)	Cover Yes	0.003	0.412	0.995
Distance to the Shed		−0.015	0.005	0.003 *
Year (Ref. Level: Year 2022)	2023	0.338	0.072	<0.001 *
Year (Ref. Level: Year 2022)	2024	−0.145	0.062	0.020 *
Cover: Distance	Cover Treated	0.000	0.003	0.870
Cover: Distance	Cover Yes	0.008	0.006	0.185
Cover: Year	Cover Treated: 2023	0.428	0.026	<0.001 *
Cover: Year	Cover Yes: 2023	−0.324	0.036	<0.001 *
Cover: Year	Cover Treated: 2024	0.327	0.026	<0.001 *
Cover: Year	Cover Yes: 2024	−0.257	0.036	<0.001 *
Distance: Year	2023	0.004	0.001	<0.001 *
Distance: Year	2024	0.008	0.001	<0.001 *
**Smooth Terms**	**Estimated df**			**Approx.** **Significance**
Temperature	21.25			<0.001 *
Q.Nr (Quadrant ID)	160.64	-	-	<0.001 *
Time: Year 2022	54.33			<0.001 *
Time: Year 2023	35.01			<0.001 *
Time: Year 2024	53.49			<0.001 *

* Indicates a significant effect with *p* < 0.05.

**Table 2:** Estimated model coefficients for the model with the number of hens per quadrant as the dependent variable for outdoor range 2 (residual df = 155,381) [26].

**Fixed Effects**	**Factor Level**	**Estimate**	**Std. Error**	***p*-Value**
Intercept	-	0.660	0.550	0.223
Cover (Ref. Level: Cover No)	Cover Treated	0.388	0.213	0.068
Cover (Ref. Level: Cover No)	Cover Yes	1.341	0.206	<0.001 *
Distance to the Shed		−0.030	0.007	<0.001 *
Year (Ref. Level: Year 2022)	2023	−0.670	0.036	<0.001 *
Year (Ref. Level: Year 2022)	2024	−0.332	0.032	<0.001 *
Cover: Distance	Cover Treated	−0.007	0.002	0.003 *
Cover: Distance	Cover Yes	−0.009	0.003	0.005 *
Cover: Year	Cover Treated: 2023	0.020	0.045	0.645
Cover: Year	Cover Yes: 2023	0.016	0.032	0.607
Cover: Year	Cover Treated: 2024	0.182	0.042	<0.001 *
Cover: Year	Cover Yes: 2024	0.309	0.031	<0.001 *
Distance: Year	2023	0.012	0.000	<0.001 *
Distance: Year	2024	0.007	0.000	<0.001 *
**Smooth Terms**	**Estimated df**			**Approx.** **Significance**
Temperature	14.21			<0.001 *
Q.Nr (Quadrant ID)	213.67	-	-	<0.001 *
Time: Year 2022	31.62			<0.001 *
Time: Year 2023	28.56			<0.001 *
Time: Year 2024	24.63			<0.001 *

* Indicates a significant effect with *p* < 0.05.

## Data Availability

The data supporting the reported results are available upon request from the corresponding author.

## Supplementary Materials

The following supporting information can be downloaded at https://www.mdpi.com/article/10.3390/ani15091204/s1, Figure S1: Aerial view of the grid on outdoor range 1. Figure S2: Smoothing terms for outdoor range 1 (a) Time (Year: 2022), (b) Time (Year: 2023), (c) Time (Year: 2024) and (d) Temperature. Figure S3: Smoothing terms for outdoor range 2 (a) Time (Year: 2022), (b) Time (Year: 2023), (c) Time (Year: 2024) and (d) Temperature. Figure S4: An example of how the cameras recording the images were mounted and orientated to different directions to cover all parts of the outdoor ranges.

## Appendix A

### Appendix A.1. Automated Data Evaluation

An automated system was developed to detect hens on the outdoor ranges and assign them to the predefined grid quadrants. The system integrated deep learning techniques combined with image processing methods and was implemented using Python (Version 3.10) [60]. The images captured by the cameras served as the primary data source for hen detection. The embedded metadata (EXIF data) in these digital images provided additional essential information such as date and time.

### Appendix A.2. Preprocessing of Grid Data

Using the open-source tool from https://www.image-map.net/ (accessed on 2 October 2023) and the digital images from the cameras, the grid quadrants on the outdoor ranges were digitally transferred to image maps provided in HTML format. The resulting polygons were named according to the unique ID of the initially defined quadrants. To simplify handling and processing, a parser was developed to extract the relevant grid coordinates from the HTML file and convert them into a JSON format suitable for further processing.

### Appendix A.3. Model Training

The YOLO large model [35] was utilised as a base model for hen detection. To optimise the model’s performance for this specific application, it was fine-tuned using a custom dataset tailored to the research objectives. This dataset comprised 371 images randomly chosen from the dataset of images collected in the current study, across both farms and different times of the day. Each image was annotated with bounding boxes around hens using the Roboflow (Version 1.0) platform [61], ensuring high-quality and consistent labelling. This annotated dataset was divided into training and validation sets using an 80/20 split, resulting in 297 images for training and 74 images for validation.

To enhance the model’s ability to learn spatial features and improve detection accuracy, especially for small and distant hens, each training image was segmented into a 10 × 10 grid of smaller patches. This gridding strategy effectively increased the number of training samples and introduced variability in spatial configurations, enabling the model to better recognise hens at significant distances. Additionally, data augmentation techniques were applied, including mosaic augmentation and lighting adjustments, to enhance the model’s robustness against variations in environmental conditions and object orientations.

All images were resized to a standardised input size of 640 × 640 pixels to balance computational efficiency with detection precision. The training process spanned 50 epochs, incorporating an early stopping mechanism to prevent overfitting. A batch size of 16 was employed and the optimiser settings adhered to the default configuration of the YOLO [35] framework. The learning rate was initialised at 0.01 and a weight decay parameter of 0.0005 was set to regulate the model.

### Appendix A.4. Image Processing and Object Detection

For the detection of hens, the fine-tuned YOLO [35] model was applied to high-resolution digital images. Given the presence of small and distant hens, each image was segmented into a 10 × 10 grid of smaller image tiles with a 20% overlap between adjacent image tiles. This tiling strategy facilitated efficient processing and enhanced the model’s ability to detect hens occupying smaller regions of the image due to their distance from the camera.

Each tile was individually processed by the YOLO [35] model, which generated bounding boxes for potential hen detections within that segment. To mitigate duplicate detections across overlapping tiles, Non-Maximum Suppression (NMS) was implemented with an Intersection over Union (IoU) threshold of 0.3, chosen empirically to balance duplication and missed detections. This deduplication process effectively reduced redundant bounding boxes, ensuring that each hen was counted only once and thereby improved the overall detection accuracy.

### Appendix A.5. Position Determination and Grid Assignment

The bottom centre point of each hen bounding box was selected as the representative location, as this point most accurately reflected the hen’s actual position on the ground. The coordinates of these points were then compared with the predefined grid polygons using geometric operations provided by the Shapely (Version 2.0.6) library [62] to determine the grid assignment for each hen. The number of hens within each grid quadrant was counted to provide quantitative data on their spatial distribution on the outdoor ranges.

### Appendix A.6. Temporal Assignment

Additionally, the date and time the digital images were taken were extracted from the EXIF metadata embedded in each digital image. These temporal data (divided into year, month, day, hour and minute) were then stored in a structured CSV file alongside the quantitative data of counted hens per quadrant. This allowed for the temporal analysis of hen distribution patterns.

### Appendix A.7. Result Visualisation

To verify and present the findings, the detected hens and grids were visualised on the original images. Detected hens were annotated with red bounding boxes and grid quadrants were outlined in red. Additionally, quadrant IDs were displayed using appropriate font sizes at the centroid of each grid. These annotated images were saved in a separate directory to ensure that the original images remained unaltered.

## Appendix B

### Appendix B.1. Final Model: Outdoor Range 1

gam_nb_farm1 <- gam(Number_of_Hens ~ Cover×Distance + Cover×Year + Distance×Year + offset(log(Size_Parts)) + s(Temperature, bs = “cr”, k = 25) + s(Q.Nr, bs = “mrf”, k = 171, xt = list(“penalty” = nb_farm1_shp_m)) + s(Time, by = Year, bs = “cr”, k = 73), data=data_farm1, family=nb())

### Appendix B.2. Final Model: Outdoor Range 2

gam_nb_farm2 <- gam(Number_of_Hens ~ Cover×Distance + Cover×Year + Distance×Year + offset(log(Size_Parts)) + s(Temperature, bs = “cr”, k = 19) + s(Q.Nr, bs = “mrf”, k = 225, xt = list(“penalty” = nb_farm2_shp_m)) + s(Time, by = Year, bs = “cr”, k = 73), data=data_farm2, family=nb())
