# Supplementary material for: Enhancing Range Use in Free-Range Laying Hen Systems: The Impact of Vegetation Cover over Time
Source: Animals (Basel). 2025 Apr 23;15(9):1204. doi: 10.3390/ani15091204 (PMC12071058; doi:10.3390/ani15091204)
Supplement: Supplementary file 1 [file animals-15-01204-s001.zip › animals-3559225-supplementary.pdf]

Supplementary Material

Enhancing Range Use in Free-Range Laying Hen Systems: The Impact of Vegetation Cover Over Time

Markus Pacher-Deutsch, Philipp Meyer, Harald Meimberg and Martin Gierus

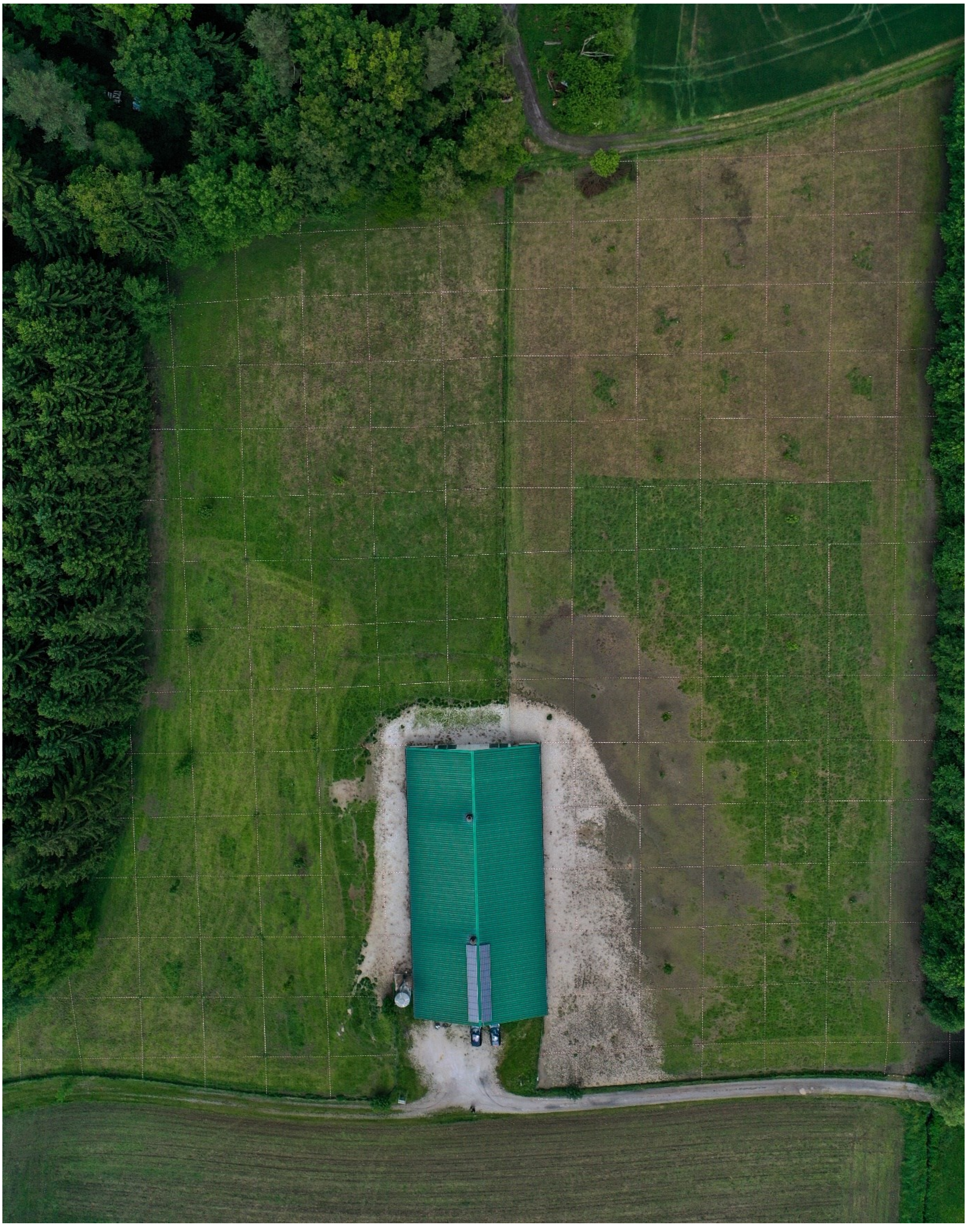

Figure S1. Aerial view of the grid on outdoor range 1

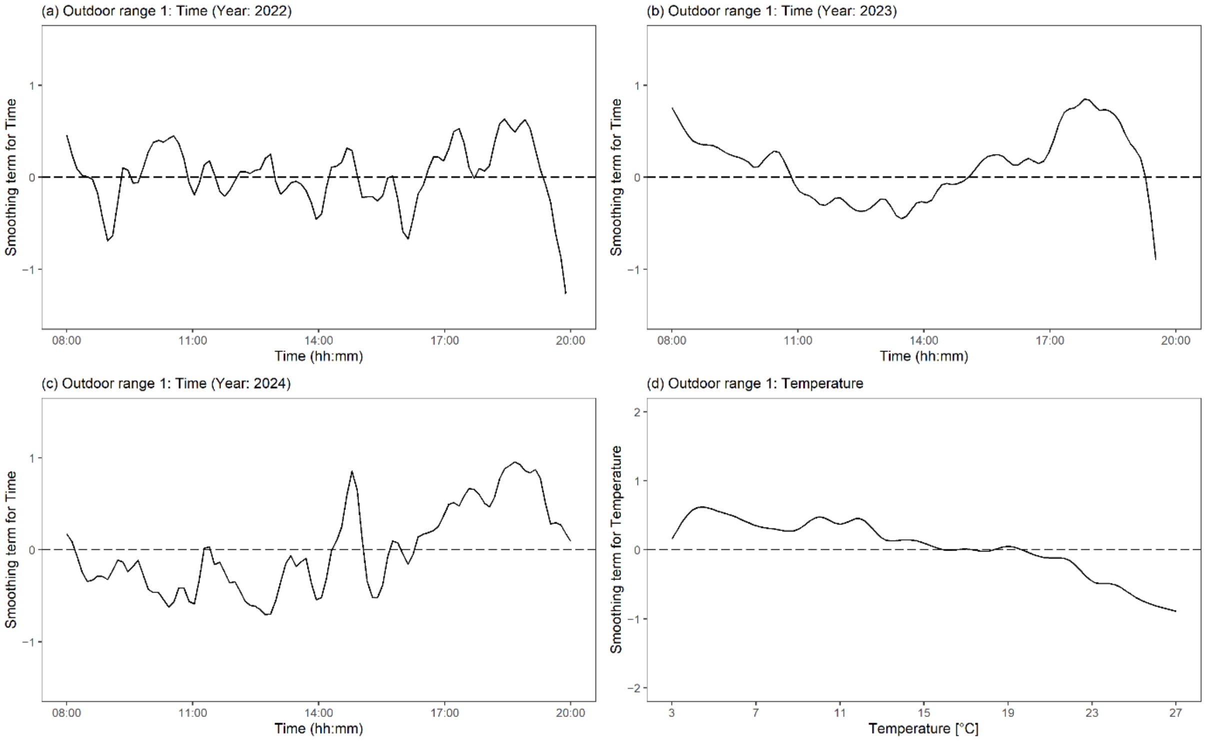

Figure S2. Smoothing terms for outdoor range 1 (a) Time (Year: 2022), (b) Time (Year: 2023), (c) Time (Year: 2024) and (d) Temperature.

In Year 0 (2022, Figure S2a) and Year 2 (2024, Figure S2c), more or less regular fluctuations in the expected number of hens on outdoor range 1 can be seen during the day. This probably corresponds to the feeding times when feeding machines inside the shed went on and therefore hens went inside the shed. In Year 1 (2023, Figure S2b) however, a diurnal pattern can be seen more clearly, with more hens than expected on the outdoor range in the morning and evening hours, but with less hens than expected during midday (this is in line with the heatmaps of outdoor range 1, where the lowest number of hens in all three years was observed during midday hours in 2023).

With increasing temperatures (e.g., during midday and afternoon hours), the expected number of hens on the outdoor range decreases (Figure S2d), which may further indicate the importance of shelter and shade offering vegetation cover on the outdoor range.

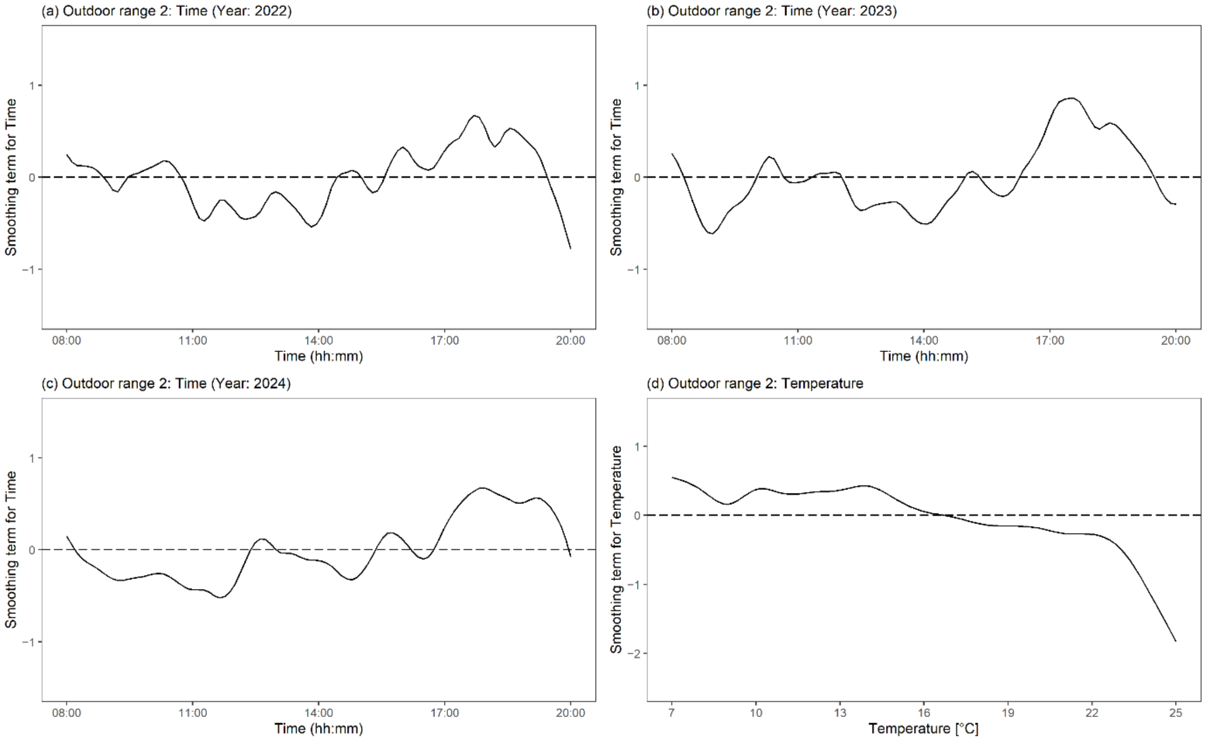

Figure S3. Smoothing terms for outdoor range 2 (a) Time (Year: 2022), (b) Time (Year: 2023), (c) Time (Year: 2024) and (d) Temperature.

As on outdoor range 1, certain fluctuations can be seen in the expected number of hens on outdoor range 2 throughout the day. This again corresponds to the feeding times inside the shed. In all three years, more hens than expected visit the outdoor range in the evening hours, whereas on average less hens than expected visit the outdoor range during midday hours. This effect occurs especially in Year 0 (2022, Figure S3a), whereas in Year 1 (2023, Figure S3b) and Year 2 (2024, Figure S3c) this effect is not that strong. This could be due to the fact, that the increased level of vegetation cover attracts more hens to the outdoor range during the day.

The expected number of hens decreases with increasing temperatures also on outdoor range 2 (Figure S3d). This strengthens the assumption that the temperature effect is not exclusive to outdoor range 1 and that temperature in general is a considerable factor when it comes to range use. Vegetation cover on the outdoor ranges would offer more shaded areas during higher temperatures, potentially leading to higher numbers of hens on the outdoor range during these hours of the day.

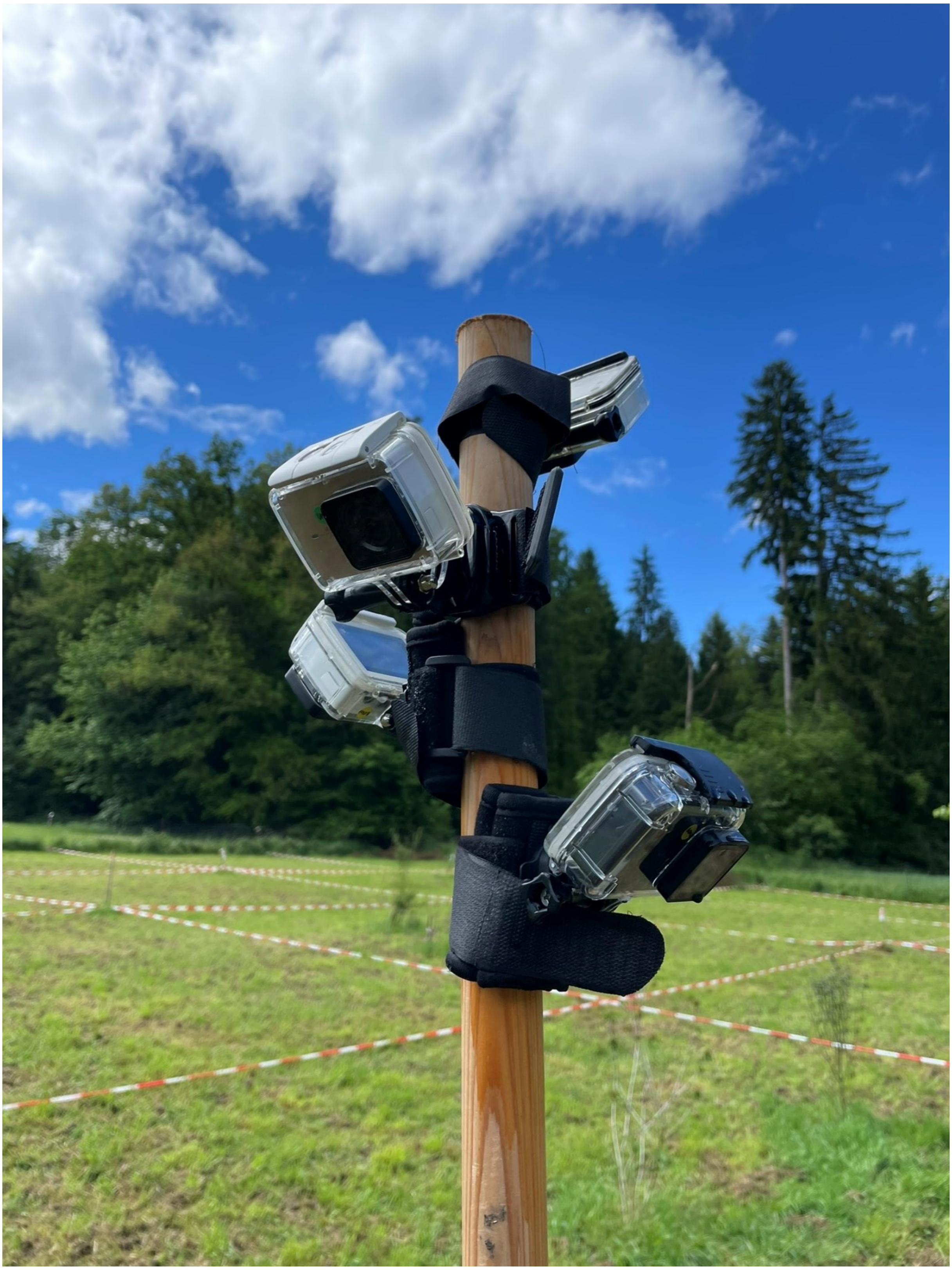

Figure S4: An example of how the cameras recording the images were mounted and orientated to different directions to cover all parts of the outdoor ranges.
